# Supplementary material for: One-Pot Self-Assembly of Core-Shell Nanoparticles within Fibers by Coaxial Electrospinning for Intestine-Targeted Delivery of Curcumin
Source: Foods. 2023 Apr 12;12(8):1623. doi: 10.3390/foods12081623 (PMC10137979; doi:10.3390/foods12081623)
Supplement: Supplementary file 1 [file foods-12-01623-s001.zip › foods-2315394-supplementary.pdf]

Supporting Material for

One-pot self-assembly of core-shell nanoparticles within fibers by coaxial electrospinning for intestine-targeted delivery of curcumin

Lijuan Hou[1], Laiming Zhang[1], Chengxiao Yu[1], Jianle Chen[1], Xingqian Ye[12], Fuming Zhang[3], Robert J. Linhardt[3], Shiguo Chen[12], Haibo Pan[12*]

[1] College of Biosystems Engineering and Food Science, National-Local Joint Engineering Laboratory of Intelligent Food Technology and Equipment, Zhejiang Key Laboratory for Agro-Food Processing, Integrated Research Base of Southern Fruit and Vegetable Preservation Technology, Zhejiang International Scientific and Technological Cooperation Base of Health Food Manufacturing and Quality Control, Zhejiang University, Hangzhou 310058, China;

[2] Innovation Center of Yangtze River Delta, Zhejiang University, Jiaxing 314102, China;

[3] Center for Biotechnology and Interdisciplinary Studies, Rensselaer Polytechnic Institute, Troy, New York, USA.

* Correspondence: apanhaibo@126.com.

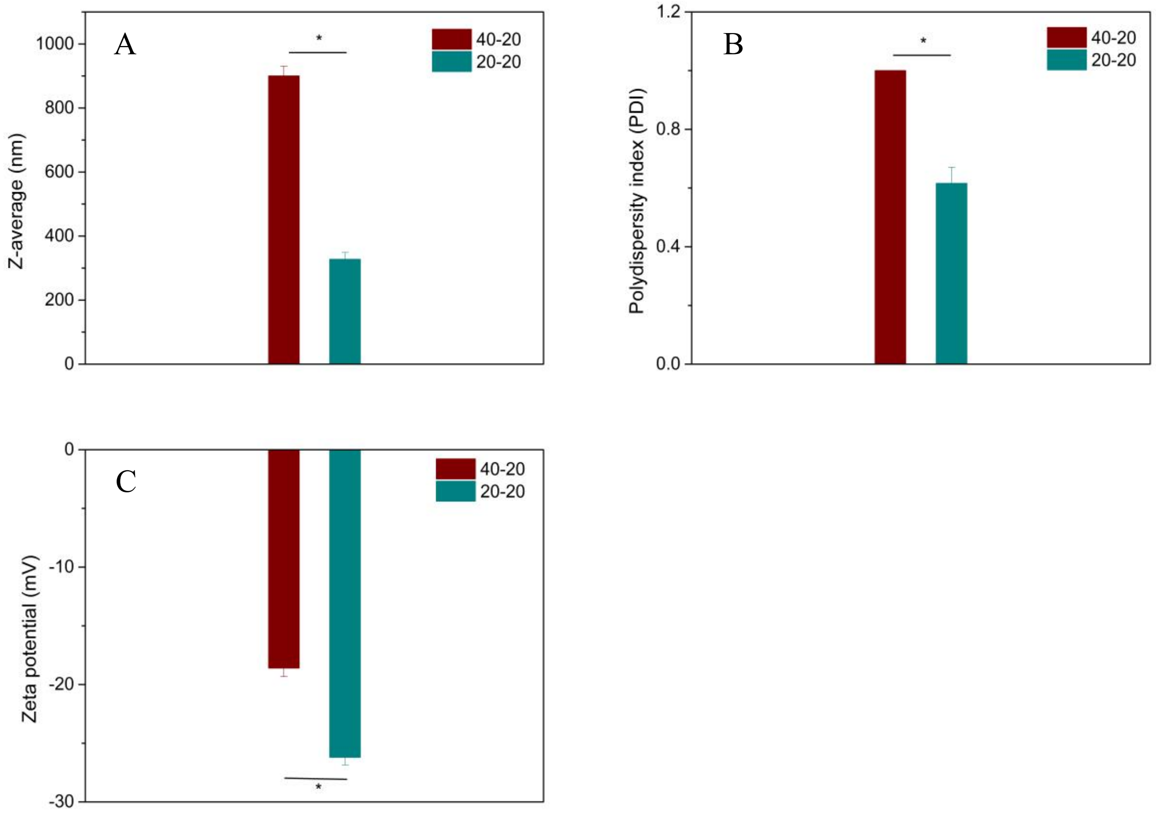

Figure S1. (A) Z-average (average particle size), (B) Polydispersity index (PDI), (C) Zeta potential of released self-assembled Z-Cur (20-20) and Z-Cur (40-20) NPs. *Indicates significant differenced as estimated by Tukey-tests ($p<0.05$), and error bars represent the standard deviation.

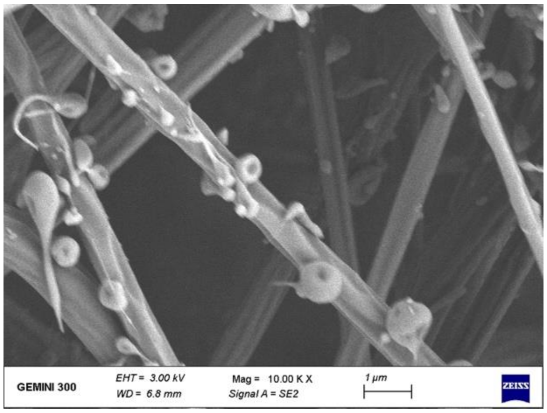

Figure S2. SEM of electrospinning ES100-Z-Cur with Z-Cur (40-20).
